# Extrafacial involvement in lupus miliaris disseminatus faciei

**DOI:** 10.1016/j.jdcr.2026.04.046

**Published:** 2026-05-08

**Authors:** Julia A. Giordano, Tatiana Zuluaga, Rosalie E. Elenitsas, Avrom S. Caplan, Misha Rosenbach

**Affiliations:** a Perelman School of Medicine, University of Pennsylvania, Philadelphia, Pennsylvania; b NYU Grossman School of Medicine, New York, New York; c Department of Dermatology, Perelman School of Medicine, University of Pennsylvania, Philadelphia, Pennsylvania; d The Ronald O. Perelman Department of Dermatology, NYU Grossman School of Medicine, New York, New York

**Keywords:** biologic therapy, extrafacial involvement, granulomatous dermatoses, intralesional corticosteroids, laser therapy, lupus miliaris disseminatus faciei, refractory inflammatory skin disease, scarring prevention

A woman in her 40s presented with a 2-month history of inflammatory papules on the philtrum, melolabial folds, left eyebrow, and axilla (Fig 1, A). Histopathology revealed a dermal infiltrate of epithelioid histiocytes surrounding central necrosis, findings most consistent with lupus miliaris disseminatus faciei (LMDF) (Fig 2). Initial treatment with prednisone 5 mg daily (prescribed externally) for 1 month, topical tacrolimus 0.1%, hydroxychloroquine 200 mg daily, and doxycycline 100 mg twice daily yielded incomplete response. The patient had concerns over isotretinoin and experienced fatigue with dapsone, so given persistent disease at 6 months, adalimumab 40 mg weekly was added to her regimen. After 4 months, incomplete improvement prompted addition of oral dapsone 50 mg daily and topical ruxolitinib. While oral dapsone provided clinical benefit, treatment was complicated by anemia at 50 mg daily, necessitating reduction to 25 mg daily. Hydroxychloroquine was subsequently discontinued to allow continuation of the highest tolerable dapsone dose. Topical ruxolitinib was discontinued due to lack of efficacy, and topical dapsone was initiated with limited effect. Intralesional triamcinolone injections were administered intermittently for flares with significant benefit. Seven months after dapsone initiation, the patient achieved complete clearance on combination therapy with dapsone alternating 25-50 mg daily, doxycycline 100 mg daily, and adalimumab 40 mg weekly, with planned gradual tapering of therapy (Fig 1, B).

**Question: Which of the following statements regarding LMDF is most accurate?**

**A.** LMDF is a self-limited disorder restricted to the periocular region.
**B.** The presence of central necrosis on histopathology favors sarcoidosis over LMDF.
**C.** LMDF may involve nonperiocular facial and extra-facial sites.
**D.** Topical therapies alone are typically sufficient to achieve disease control in LMDF.
**E.** Laser therapy is absolutely contraindicated in LMDF.

## Discussion

LMDF traditionally presents as papules on the central face, classically around the eyelids, and has historically been described as a periocular condition.^1^ However, accumulating evidence, including our case, reinforces that LMDF may frequently involve other facial and extra-facial sites, including the chin, cheeks, perioral region, and axillae.^1^, ^2^, ^3^

Recognition of these distributions is important, as chin and perioral papules may closely resemble perioral dermatitis, while axillary lesions may mimic hidradenitis, sarcoidosis, or folliculitis. LMDF may overlap clinically and histopathologically with granulomatous rosacea; however, distinguishing features of LMDF include the presence of caseating granulomas, absence of classic rosacea features, and involvement of extrafacial sites such as the axilla. Clinicians should closely examine perioral, chin, cheek, and axillary regions, particularly when orange-brown papules or papulonodules are observed, to facilitate early diagnosis and mitigate scarring potential.^1^, ^2^, ^3^

Therapeutically, LMDF remains challenging to manage. A range of treatments have been reported with variable efficacy, including tetracycline antibiotics, isotretinoin, dapsone, hydroxychloroquine, systemic corticosteroids, and calcineurin inhibitors.^1^, ^2^, ^3^, ^4^, ^5^ Biologic agents such as adalimumab may be considered for refractory cases.^4^ Gradual tapering may be attempted after disease control. Given the expanded use of JAK-inhibitors for granulomatous diseases, this class of drugs may be explored in the future for recalcitrant cases.

Intralesional triamcinolone and laser therapy appear promising adjunctive treatments. Recent reports suggest that laser-based modalities, including PDL and fractionated CO2 laser, can provide symptomatic relief and improve scarring.^5^ Caution is advised when employing laser on active granulomatous inflammation due to rare risks of worsening or ulceration.

This case highlights several important teaching points. First, LMDF may present in underrecognized anatomic locations beyond the periocular region, including the perioral area and axillae. Second, refractory LMDF often necessitates combination systemic therapy, including biologic agents. Third, adjunctive laser therapy may provide benefit in selected patients. Awareness of these nuances can facilitate earlier recognition and optimize outcomes in patients with this condition.

**Fig 1:**
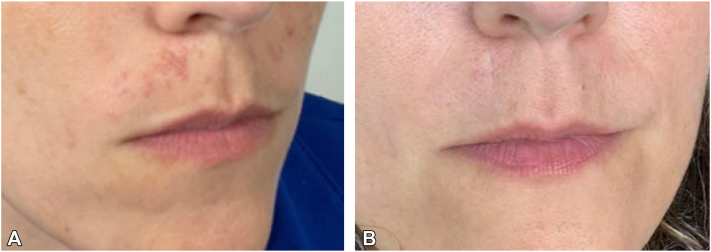
**A,** Patient on initial presentation, demonstrating inflammatory papules on the philtrum and melolabial folds. **B,** Patient after combination therapy treatment with doxycycline, dapsone, and adalimumab.

**Fig 2:**
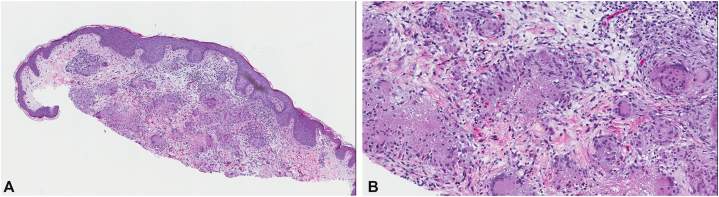
**A,** Shave biopsy from the axilla reveals multiple granulomas in the dermis. (hematoxylin and eosin, ×60). **B,** Epithelioid histiocytes and multinucleated giant cells surround an area of necrosis. (hematoxylin and eosin, ×180).

## Conflicts of interest

None disclosed.
